# Psychosis and concurrent impulse control disorder in Parkinson's disease: A review based on a case report

**DOI:** 10.1590/S1980-5764-2016DN1002011

**Published:** 2016

**Authors:** Bruno Fukelmann Guedes, Marcia Rubia Gonçalves, Rubens Gisbert Cury

**Affiliations:** 1,2 MD, Department of Neurology, Hospital das Clínicas - University of São Paulo, São Paulo SP, Brazil.; 3 MD PhD, Department of Neurology, Hospital das Clínicas - University of São Paulo, São Paulo SP, Brazil.

**Keywords:** impulse control disorders, Parkinson's disease, psychotic disorders, punding, gambling

## Abstract

Psychosis, impulse control disorders (e.g., pathological gambling and hypersexuality) and repetitive behaviors such as punding are known psychiatric complications of Parkinson's disease (PD). Impulsive, compulsive and repetitive behaviors are strongly associated with dopamine-replacement therapy. We present the case of a 58-year-old man with PD and a myriad of psychiatric symptoms. Concurrent psychosis, punding and pathological gambling developed more than six years after the introduction of pramipexole and ceased shortly after the addition of quetiapine and discontinuation of pramipexole. This report emphasizes the importance of monitoring for a wide array of psychiatric symptoms in patients on dopamine replacement therapy.

## RESUMO

Psicose, transtornos do impulso (como jogo patológico e hipersexualidade) e comportamentos repetitivos como *punding* são complicações psiquiátricas conhecidas da doença de Parkinson. Comportamentos repetitivos, impulsivos e compulsivos são fortemente associados a terapia de reposição dopaminérgica. Nós apresentamos o caso de um homem de 58 anos com doença de Parkinson de início precoce e múltiplos sintomas psiquiátricos. Psicose, *punding* e jogo patológico surgiram mais de seis anos após a introdução de pramipexol e cessaram pouco após sua descontinuação e introdução de quetiapina. Nosso relato enfatiza a importância do monitoramento para uma ampla gama de sintomas psiquiatricos em pacientes com doença de parkinson em uso de terapia de reposição dopaminérgica.

## INTRODUCTION

Neuropsychiatric symptoms are common in Parkinson's disease (PD) and encompass a wide range of disturbances such as anxiety, apathy, depression, hallucinations, delusions, irritability and apathy.^1^ ^,^ ^2^ Delirium and depression have long been thought to be associated with PD.^3^ However, in the era of polypharmacological treatment with dopamine-replacement therapy, the spectrum of behavioral symptoms of PD has been broadened. Psychosis and impulsive behavior have emerged as important complications of the disease and its treatment. These psychiatric symptoms usually begin within a few weeks of introducing or increasing antiparkinsonian drugs, especially dopaminergic agonists. We report the case of a patient with atypical presentation that developed concurrent psychosis, punding and pathological gambling after long-term use of a stable dose of antiparkinsonian drugs with good outcome following medication adjustment. The objective of the present study was to highlight this uncommon presentation and review some of the epidemiological and clinical aspects of these major psychiatric disorders associated with PD.

## CASE REPORT

A 58-year-old man was referred to a tertiary center for marked psychiatric symptoms and worsening parkinsonism without medical comorbidities. The patient started to exhibit parkinsonism at the age of 46, when he developed generalized slowing and asymmetric resting tremor of the extremities, which evolved to impairments in balance and gait over time. He was diagnosed with Parkinson's disease on the basis of clinical findings and on his response to dopamine-replacement therapy. He was started on an increasing pramipexole dosage up to 1.5mg TID and levodopa (initially at low doses). He had no comorbidities. 

Four years after the onset of symptoms, the patient developed motor complications, dyskinesia, and gait freezing. When the patient was 52 years old, he developed a pathological gambling habit. He had played cards with friends since the age of 20 with occasional, non-significant losses. He would play twice a week, and the gambling habit had never been disruptive. With the worsening of his symptoms, the patient's gambling habits changed. He switched from cards to slot machines and started gambling alone. He would play daily and throughout the night, losing increasing amounts of money. Within a few months, his family's income was compromised.

At the same time, the patient developed a stereotypical, unique behavior. He began collecting rubbish and miscellaneous items of litter; his family described the patient as obsessed. He stockpiled trash at home and would collect and assemble different parts of broken machinery. He repeatedly stated that he would "make working things out of the parts collected", and claimed that this work was a potential business. However, the patient never actually fixed any of these devices. He started to spend hours and sometimes days dedicated solely to this behavior. He skipped meals and lost weight. He was partially cognizant of the inappropriateness of his behavior.

Notably, these impulsive symptoms were not associated with any change in the dosage of pramipexole or levodopa. Neither the patient nor the caregivers regarded the impulsive behavior as being related to the patient's underlying disease, which may explain why the behavior was not reported to his treating physician at the time.

Five months after the onset of the impulsive behavior, the patient developed psychosis. He had paranoid delusions and became suspicious of his relatives and caregivers. After three months, he started to hear voices that ordered him around. The voices said the patient's mother was a witch and ordered him to kill her. The patient struck his mother with a broomstick and was admitted to a neurology clinic. His treatment regimen was changed; quetiapine was introduced and titrated up to 200mg BID with complete resolution of psychotic symptoms after 2 weeks. After a few months, his daily levodopa dose was lowered from 600mg to 350mg and pramipexole was discontinued. Impulsive symptoms improved significantly.

Less than 5 months after the patient's treatment was adjusted, he was evaluated at our clinic. The patient reported complete resolution of his gambling, litter-related behavior and psychosis, although his Parkinsonism had significantly worsened. He exhibited no signs or symptoms of depression. His Unified Parkinson's Disease Rating Scale total score was 69/199 (3/16 points in behavior, 10/52 in daily activities, 50/108 in motor symptoms and 6/23 in motor complications), and his MMSE score was 26/30 (he had eight years of formal education). On the verbal fluency test, he was able to name 25 words beginning with a single letter in one minute. On the category (semantic) fluency test, he named 22 animals in 60 seconds. He performed well on the alternating sequences task. No formal neuropsychological evaluation was performed. He showed no signs of cognitive impairment, and his levodopa dosage was titrated gradually.

After a follow-up period of two months, the patient was receiving levodopa at 1100mg/day and quetiapine at 200mg BID. He exhibited marked improvement in his motor symptoms with no sign of re-emergence of behavioral symptoms.

## DISCUSSION

In this report, we described a case involving late-onset of concomitant psychosis and ICD in a patient in long-term use of stable doses of dopamine agonist (DA). Psychotic symptoms are rare in untreated patients with PD, and appear to be associated with DA therapy, cognitive impairment and depression.^4^ DAs have specific affinity for D3 and D2 receptors. D3 receptors mediate motor responses, but are also located in limbic nuclei, and are a target for psychiatric drugs. D3 receptor stimulation has also been implicated in the addiction process. Thus, DA stimulates pathways that are related to addiction and pleasure, and can provoke behavioral alterations (psychosis and ICD).^5^ In a single-center study of 289 patients, Pacchetti et al.^6^ found psychotic symptoms, classified as either delusions or hallucinations, in 32% of patients. Consistent with the high prevalence of visual hallucinations found in patients with dementia with Lewy bodies {Møller:2013ieba}, isolated visual hallucinations were the most common presentation. Only 17.4% of patients had auditory hallucinations. Interestingly, paranoid psychosis was identified in 14% of patients with psychotic symptoms (4% of total study population), showing that this manifestation may not be uncommon in PD.^6^

Addressing psychosis in PD may be challenging because treatment strategies often require discontinuation of dopamine-replacement therapy and introduction of antipsychotics, which may worsen motor symptoms, as shown in our case. After excluding medical causes of delirium, an initial strategy would be discontinuation of non-levodopa medications and patient surveillance to monitor for motor deterioration. Antipsychotics may be used with caution; those antipsychotics with strong D1 and D2 antagonism (olanzapine, risperidone) have been shown to worsen Parkinsonism.^7^ ^,^ ^8^ There is consistent evidence supporting the use of clozapine to control psychosis without motor deterioration. However, in resource-limited settings, widespread use of clozapine is precluded because of the need to carry out close monitoring for adverse hematological events. Despite the lack of literature evidence regarding the use of quetiapine for psychiatric symptoms associated with PD, it is used off-label as a first-line agent in many centers because it can be used without specialized monitoring.^9^

Impulse control disorders (ICD) are a class of psychiatric disturbances that share the core feature of failure to resist a temptation to engage in behaviors that are harmful to oneself or others.^10^ Classical ICDs include pathological gambling, compulsive sexual behavior and compulsive buying. Although punding has some differences from these classical ICDs, it involves the same essential characteristics. ICDs and punding, along with dopamine dysregulation syndrome, may be considered a broader class of impulse control and repetitive behavior disorders (ICRBs).

ICDs such as pathological gambling are not associated with PD itself, but rather with dopamine-replacement therapy,^11^ mostly DAs. Data suggests that risk factors for ICD in PD can include younger age or younger age at PD onset, male sex, pre-PD history of ICD symptoms (the present patient had a history of gambling), history of substance abuse or bipolar disorder, and personality style characterized by impulsiveness.^12^ ICDs can occur at any time during treatment with DAs.^13^ Incidence seems to be most common within a few months of initiating or increasing the DA dose.^13^ ^,^ ^14^ Since ICDs are strongly associated with DA therapy, many propose DA dosage adjustment as the mainstay treatment. This recommendation is supported by several case reports and case series.^11^ In the present case, the addition of low-dose quetiapine led to complete remission of psychosis but had no effect on ICRBs. It was not until quetiapine was titrated to 200mg BID and pramipexole discontinued that the patient achieved complete remission. As mentioned above, the use of quetiapine in PD for ICD is still under debate, and no major evidence supporting its efficacy in psychosis and impulse disorder symptoms has been reported in the literature. Therefore, the improvement of the symptoms in the present case can only be explained by the reduction in the DA.^15^

Punding was initially described in amphetamine users by Rylander.^16^ Punding consists of complex stereotyped behavior characterized by fascination with purposeless and repetitive activities such as hobbyism, hoarding and continuous handling of equipment or electronics.^17^ The exact prevalence of punding is unknown, and varies across studies. Values as low as 0.34% and as high as 14% have been reported.^18^ Punding is associated with dopamine-replacement therapy, but not specifically with DAs. It is more associated with D1 agonists such as levodopa or apomorphine. Given the similarities with dyskinesias, a multistep treatment algorithm that consisted of a reduction in levodopa doses followed by withdrawal of other D1 agonists, addition of amantadine and finally, introduction of quetiapine, provided complete relief in 6/10 patients with punding.^19^ Notably, the present patient improved after an increase in daily quetiapine dose and discontinuation of pramipexole. His remission persisted even though levodopa was titrated to a significantly high total daily dose (1100mg/d).

In conclusion, psychiatric symptoms add to the complexity of Parkinson's disease. Growing evidence suggests specific dopaminergic pathways and different treatment strategies for each psychiatric syndrome. The case reported highlights the complex clinical picture of overlapping psychiatric syndromes in describing the rare association of punding, pathological gambling and psychosis.
